# Differential T Cell Signaling Pathway Activation by Tacrolimus and Belatacept after Kidney Transplantation: Post Hoc Analysis of a Randomised-Controlled Trial

**DOI:** 10.1038/s41598-017-15542-y

**Published:** 2017-11-09

**Authors:** Nynke M. Kannegieter, Dennis A. Hesselink, Marjolein Dieterich, Gretchen N. de Graav, Rens Kraaijeveld, Carla C. Baan

**Affiliations:** Department of Internal Medicine, Erasmus MC, University Medical Center Rotterdam, Rotterdam, The Netherlands

## Abstract

Pharmacokinetic immunosuppressive drug monitoring poorly correlates with clinical outcomes after solid organ transplantation. A promising method for pharmacodynamic monitoring of tacrolimus (TAC) in T cell subsets of transplant recipients might be the measurement of (phosphorylated) p38MAPK, ERK1/2 and Akt (activated downstream of the T cell receptor) by phospho-specific flow cytometry. Here, blood samples from n = 40 kidney transplant recipients (treated with either TAC-based or belatacept (BELA)-based immunosuppressive drug therapy) were monitored before and throughout the first year after transplantation. After transplantation and in unstimulated samples, p-p38MAPK and p-Akt were inhibited in CD8^+^ T cells and p-ERK in CD4^+^ T cells but only in patients who received TAC-based therapy. After activation with PMA/ionomycin, p-p38MAPK and p-AKT were significantly inhibited in CD4^+^ and CD8^+^ T cells when TAC was given, compared to pre-transplantation. Eleven BELA-treated patients had a biopsy-proven acute rejection, which was associated with higher p-ERK levels in both CD4^+^ and CD8^+^ T cells compared to patients without rejection. In conclusion, phospho-specific flow cytometry is a promising tool to pharmacodynamically monitor TAC-based therapy. In contrast to TAC-based therapy, BELA-based immunosuppression does not inhibit key T cell activation pathways which may contribute to the high rejection incidence among BELA-treated transplant recipients.

## Introduction

Pharmacokinetic monitoring of the most frequently used immunosuppressive drug after solid organ transplantation, tacrolimus (TAC), is most often based on whole-blood, pre-dose concentrations. The TAC pre-dose concentration, however, has an imperfect correlation (r_s_ ≈ 0.7) with the total exposure to TAC during a dosing interval as measured by the area-under the concentration *versus* time-curve^1–3^. As a consequence, and due to a high intra-patient variability in TAC exposure, the occurrence of acute rejection or side effects is not accurately predicted by TAC pre-dose concentrations^4–7^.

T cells are the main target of most immunosuppressive drugs used in transplantation. T cells become activated upon three separate stimulation signals: 1) antigen recognition by the T cell receptor (TCR) with the help of antigen presenting cells (APC); 2) co-stimulation, of which the interaction between CD28 molecules on T cells and CD80/86 molecules on the APC is the best known pathway, and 3) binding of cytokines^8^. This will activate intracellular signaling pathways downstream of the TCR [including the calcineurin, Mitogen-Activated Protein Kinase (MAPK) and PI3K pathways] and initiate the activation of transcription factors that regulate the production of cytokines (*e*.*g*. IL-2, IFN-γ and TNF-α)^9^. The activation of these pathways is characterized by (de-)phosphorylation of specific signaling molecules: Nuclear Factor of Activated T cells (NFAT), p38MAPK, Extracellular signal-Regulated Kinases 1 and 2 (ERK1/2) and AKT8 virus oncogene cellular homolog (Akt) (see also Supplementary Figure S1)^10–14^.

A promising approach to determine the biological effects of immunosuppressive drugs may be the measurement of intracellular signaling pathway activation^4,15–19^. Several research groups have tried to find suitable biomarkers for pharmacodynamic immunosuppressive drug monitoring, such as NFAT-regulated gene expression^20–22^. Up until now, these methods have not found their way into routine clinical practice^23–26^.

Phospho-specific flow cytometry of the intracellular signaling molecules p38MAPK, ERK and Akt is a promising technique to monitor the pharmacodynamic effects of immunosuppressive drugs in whole-blood of kidney transplant patients^27^. Previous studies on phospho-specific flow cytometry have demonstrated that TAC can inhibit p38MAPK in a dose-dependent manner in kidney transplant patients^28^. Moreover, mycophenolic acid (MPA) was been found to decrease the phosphorylation of p38MAPK and ERK1/2 *in vitro* ^29^. However, these previous studies lacked an appropriate control group that did not receive TAC and could therefore not exclude an effect of other, concomitantly used immunosuppressive drugs on T cell activation in these kidney transplant patients.

Belatacept (BELA), a fusion protein consisting of the Fc-fragment of human immunoglobulin G1 linked to the extracellular domain of human cytotoxic T-lymphocyte antigen (CTLA)-4, was approved in 2011 for the prevention of acute rejection after kidney transplantation^30–32^. It blocks the co-stimulation signal between CD80/86 and CD28 molecules (on APCs and T cells, respectively) and prevents T cell activation. In contrast to TAC, BELA is not nephrotoxic and has less metabolic side effects, although the incidence of acute rejection with a BELA-based treatment is relatively high^33,34^. The higher risk of acute rejection of patients who receive treatment with BELA has been associated with a more aggressive T cell-mediated allogeneic response^33,35–37^. One of the explanations for this phenomenon is the fact that memory CD8^+^ T cells lack CD28 expression and are not dependent on the co-stimulatory signal from CD80/86^35,38–40^.

Here, we expand on our previous work and investigated the phosphorylation status of 3 signaling proteins involved in T cell activation, namely p38MAPK, ERK and Akt, by means of phospho-specific flow cytometry to investigate whether this technique can be used as a tool for pharmacodynamic monitoring of TAC-based immunosuppression after kidney transplantation^28^. Phosphorylation was measured in blood samples of kidney transplant patients treated with either a TAC-based or a BELA-based immunosuppressive regimen as a part of a randomized controlled clinical trial^34^. Both groups received MPA and prednisolone. Because BELA only indirectly inhibits T cell activation, the BELA-treated group served as a control group in this study, circumventing a limitation of previous studies^28,29^. In contrast to previous studies, the effect of TAC and BELA was also determined in different CD3^+^ T cell subsets and at multiple time points after transplantation.

## Results

### Patient demographics and graft survival

Baseline patient characteristics and information regarding patient and graft survival are shown in Supplementary Tables S2 and S3 ^34^. In summary, three patients in the BELA group lost their graft at day 12 (Banff type IIB acute rejection), day 59 (Banff type III acute rejection) and day 161 (Banff type IIB acute rejection) after transplantation. The total incidence of biopsy-proven acute rejection (BPAR) in this group was 55% (n = 11, median time to rejection 81 days). The incidence of BPAR was lower in the TAC-treated group (n = 2, 10%, median time to rejection 56 days). In the TAC group, one patient died 294 days after transplantation, due to a traumatic head injury. For the present study, patients with a BPAR were censored from the moment of rejection onwards, because measurements of intracellular signaling pathways after the occurrence of rejection were likely to be heavily influenced by the anti-rejection therapy.

### T cell subset counts and immunosuppressive drug pre-dose concentrations

**Figure 1:**
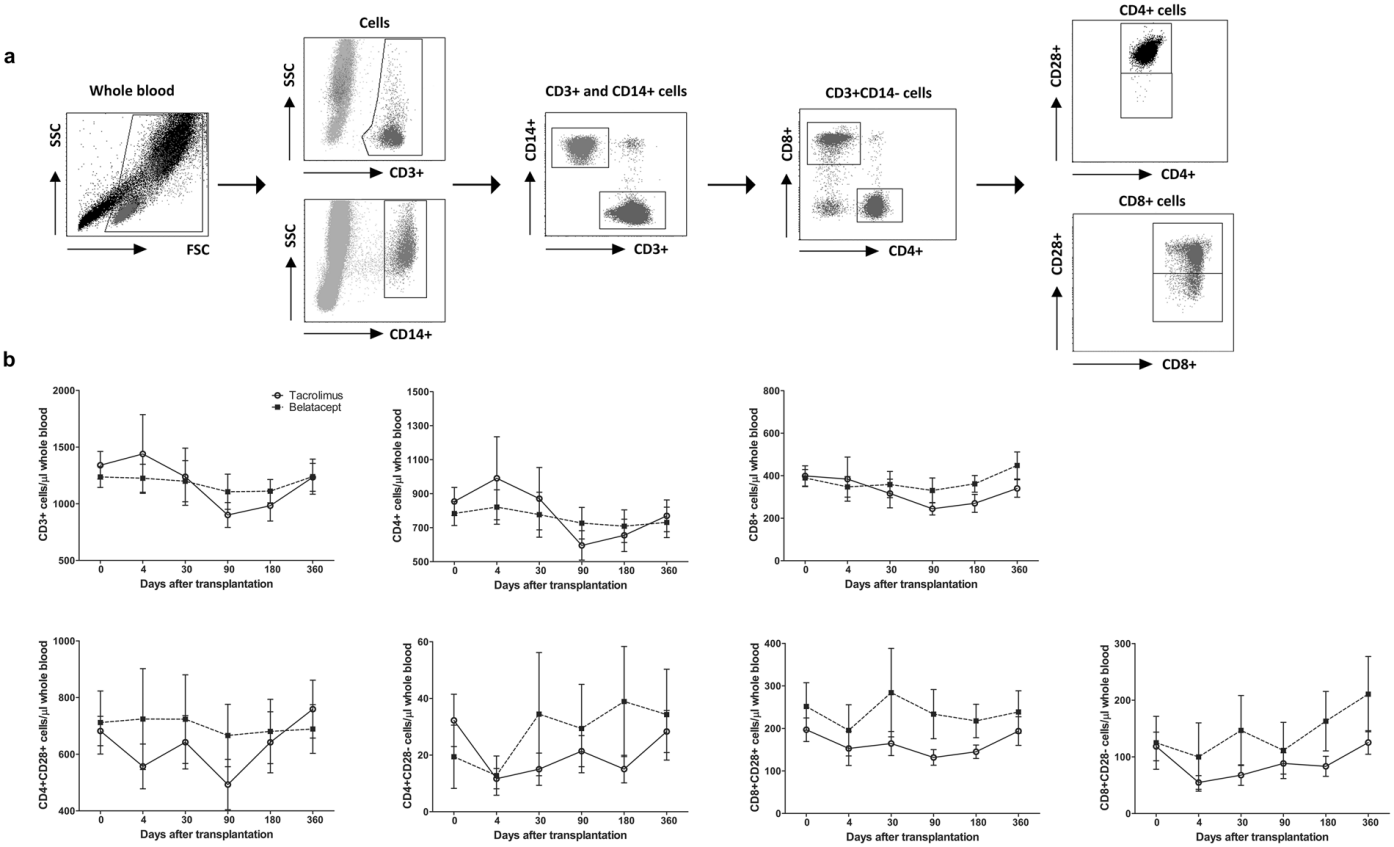
Gating strategy and T cell counts after transplantation. (**a**) Gating strategy for the phospho-specific flow cytometry assay to determine the percentages of CD3^+^ T cells and CD4^+^CD28^+^, CD8^+^CD28^+^ and CD8^ +^ CD28^−^ T cell subsets. CD3^+^ cells were selected in whole blood samples and gated for their negative expression of CD14. Then, CD3^+^CD14^−^ cells were separated for their expression of CD4 or CD8 and their expression of CD28 within these subpopulations. (**b**) Absolute numbers of CD3^+^ T cells and T cell subsets over time. TAC- (open circles) and BELA-treated (black boxes) patients showed no significant difference in T cell subset expression after transplantation. (Data are plotted as the mean ± SEM; n = 20 TAC-treated patients and n = 20 BELA-treated patients); FSC) Forward scatter; SSC) sideward scatter.

Figure 1a represents a gating strategy example of the phospho-specific flow cytometry measurements and the selection of the CD3^+^ T cell subsets. Absolute numbers of CD3^+^, CD4^+^, CD8^+^, CD4^+^CD28^+^, CD4^+^CD28^−^, CD8^+^CD28^+^ and CD8^+^CD28^−^ T cells were constant over time after transplantation (Fig. 1b). There were no differences between the absolute T cell counts of TAC- and BELA-treated patients (Fig. 1b).

TAC pre-dose concentrations decreased over time and followed the targeted pre-dose concentration range (see Supplementary Table S4). Likewise, MPA pre-dose concentrations and prednisolone doses were in the range specified by the protocol. BELA was administered according to the less-intensive regimen (10 mg/kg at days 0, 4, 30 and 90 and 5 mg/kg at days 180 and 360^34^. CD86 saturation of monocytes was complete at all time points of all BELA-treated patients^34^.

### Whole blood phospho-specific flow cytometry of p38MAPK, ERK and Akt

**Figure 2:**
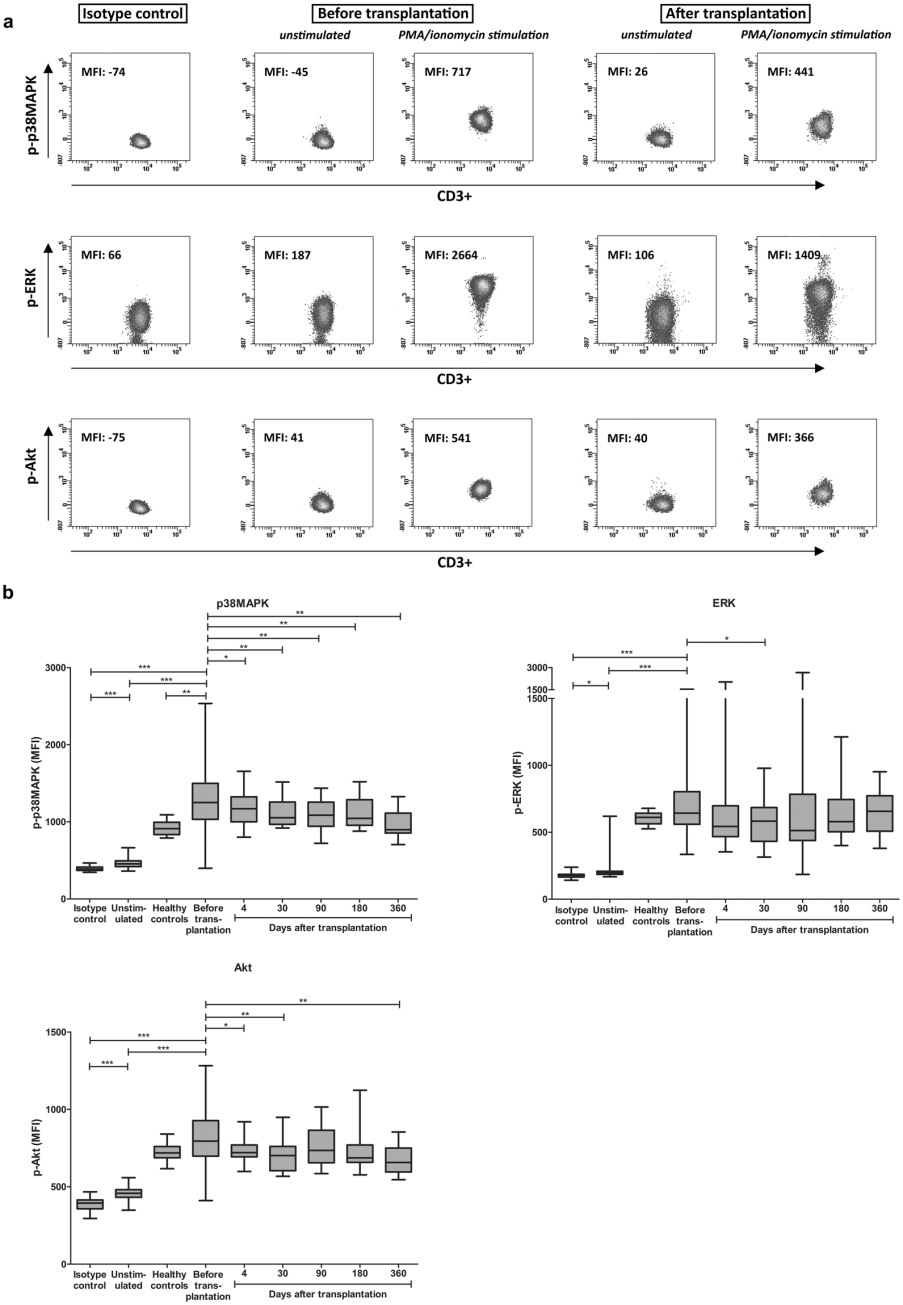
Phosphorylation of p38MAPK, ERK and Akt in CD3^+^ T cells. (**a**) Example of measured median fluorescence intensities in CD3^+^ T cells for p-p38MAPK, p-ERK and p-Akt before and after transplantation (in both unstimulated and PMA/ionomycin stimulated samples) compared to their isotype control measurements. Negative values measured with flow cytometry can be explained by the compensation settings of the FACS. (**b**) Total phosphorylation (after stimulation with PMA/ionomycin) of p38MAPK (upper left), ERK (right) and Akt (lower left) before and after transplantation compared to isotype controls, unstimulated samples and stimulated samples of healthy controls. Data are plotted as box and whiskers indicating total range. In contrast to p-ERK and p-Akt, p-p38MAPK is inhibited in CD3^+^ T cells at all time points after transplantation (n = 40); *p < 0.05, **p < 0.01, ***p < 0.001.

Phosphorylation of intracellular signaling molecules was measured with or without PMA/ionomycin stimulation to monitor the effects of the immunosuppressive drug combination therapy, consisting of either TAC or BELA in combination with prednisolone and MMF. An example of the phosphorylation measurements is depicted in Fig. 2a. In the total CD3^+^ T cell population, p-p38MAPK, p-ERK and p-Akt showed significantly higher MFI values after stimulation (before transplantation; mean MFI: 1308, 718 and 813, respectively) compared to the isotype controls (mean MFI: 394, 177 and 387, respectively; p < 0.001) and unstimulated samples (mean MFI: 466, 222 and 458, respectively; p < 0.001) (Fig. 2b). Expression of p-p38MAPK was higher in patients before transplantation than in healthy controls (mean MFI: 1308 *vs*. 916; p < 0.01). P-p38MAPK expression in CD3^+^ cells significantly decreased in the total study population and at all measured time points after transplantation, in contrast to p-ERK and p-Akt.

### Signaling protein phosphorylation in T cell subsets of TAC- and BELA-treated kidney transplant patients

**Figure 3:**
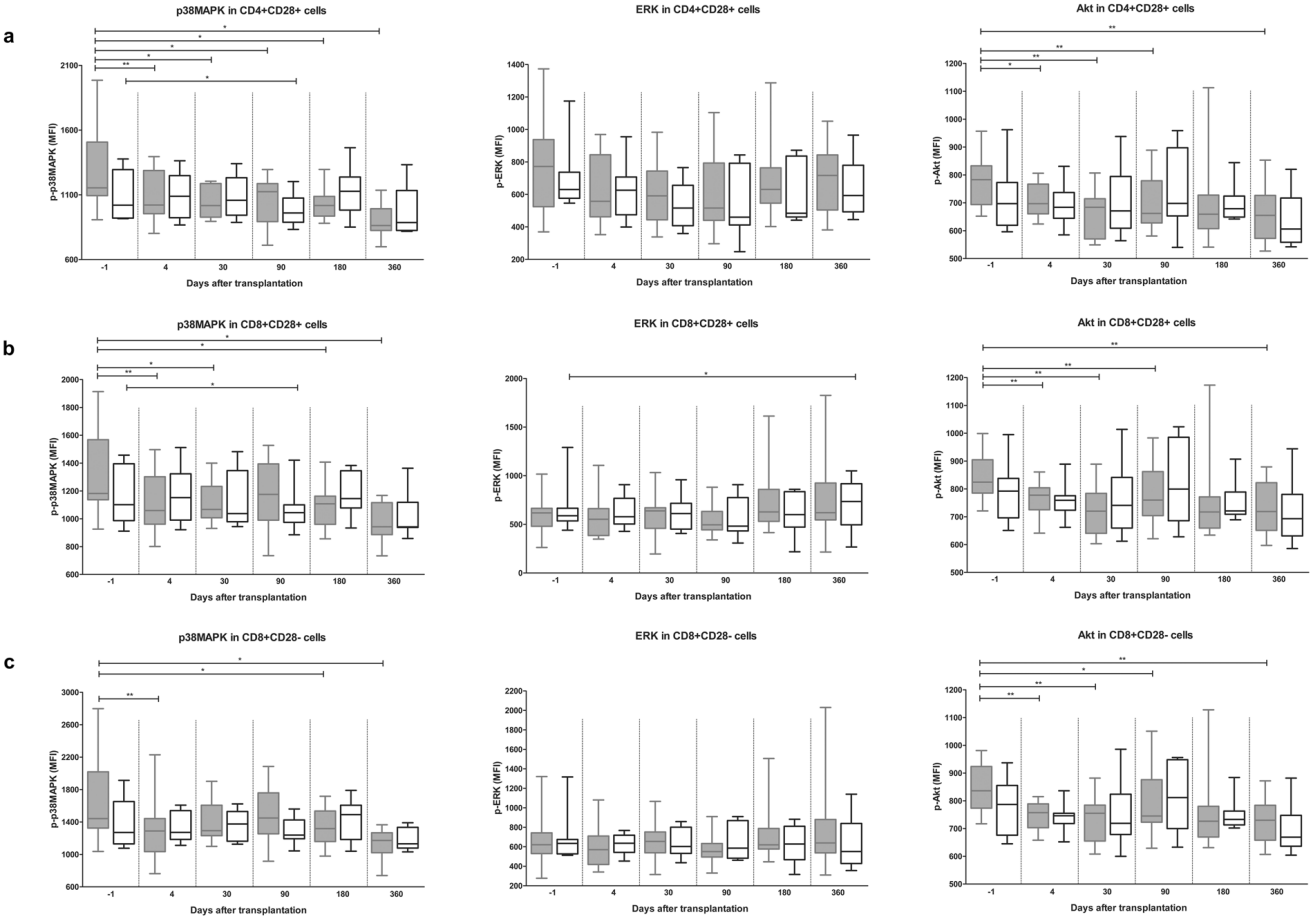
P-p38MAPK, p-ERK and p-Akt in T cell subsets of TAC- (*grey*) and BELA- (*white*) treated patients after stimulation with PMA/ionomycin. (**a**) p-p38MAPK (left), p-ERK (middle) and p-Akt (right) within CD4^+^CD28^+^ T cells. P-p38MAPK and p-Akt, but not p-ERK, were significantly decreased in TAC-treated patients at different time points after transplantation, in contrast to BELA-treated patients. (**b**) p-p38MAPK (left), p-ERK (middle) and p-Akt (right) within CD8^+^CD28^+^ T cells. P-p38MAPK and p-Akt, but not p-ERK, were inhibited when TAC was given. BELA caused an increase in p-ERK at day 360. (**c**) p-p38MAPK (left), p-ERK (middle) and p-Akt (right) within CD8^+^CD28^−^ T cells. Phosphorylation inhibition by TAC is comparable with CD8^+^CD28^+^ T cells. (Data are plotted as box and whiskers indicating total range; n = 20 TAC-treated patients and n = 20 BELA-treated patients) *p < 0.05, **p < 0.01, ***p < 0.001.

To assess the effect of TAC on T cell subset activation, phosphorylation of p38MAPK, ERK and Akt was measured before and throughout the first year after transplantation in CD4^+^CD28^+^, CD8^+^CD28^+^ and CD8^+^CD28^−^ T cell subsets of TAC-treated patients (Fig. 3 and Supplementary Figure S5). Samples of BELA-treated patients were included as controls.

In unstimulated blood samples, p-ERK was significantly inhibited in CD4^+^CD28^+^ T cells when TAC was given, but not in the presence of BELA (p < 0.01) (see Supplementary Figure S5). This phenomenon was not observed in CD8^+^CD28^+^ and CD28^−^ cells (see Supplementary Figure S5).

After PMA/ionomycin stimulation, inhibition of signaling protein phosphorylation was found for p-p38MAPK and p-Akt, but not for p-ERK and only during TAC-based treatment (Fig. 3). In CD4^+^CD28^+^ cells, TAC-based treatment showed a decreased expression of p-p38MAPK at all time points (p < 0.05) and a reduced p-Akt expression at most tested time points (p < 0.01) (Fig. 3a). In the presence of BELA, reduced expression of p-p38MAPK in CD4^+^CD28^+^ was measured only at day 90 (p < 0.05). A comparable effect of a TAC-based immunosuppressive regimen was seen in CD8^+^CD28^+^ and CD8^+^CD28^−^ T cells, although p-p38MAPK and p-Akt were not inhibited at day 90 and 180, respectively (Fig. 3b,c). Notably, at day 360, BELA-based treatment increased the expression of p-ERK in CD8^+^CD28^+^ T cells (Fig. 3b).

### Pharmacodynamic-pharmacokinetic interrelationship between immunosuppressive drug concentrations, clinical outcomes and signaling protein phosphorylation

After stimulation with PMA/ionomycin, no correlations were found between TAC pre-dose concentrations or MPA pre-dose concentrations and the phosphorylation of intracellular signaling proteins (Table 1). Also no associations were found between the phosphorylation of p38MAPK, ERK and Akt and patient characteristics pre-, 4 and 360 days after transplantation (see also Supplementary Table S6).

**Table 1:** Correlation between signaling molecule phosphorylation (stimulated) and immunosuppressive drug pre-dose blood concentrations at day 4 after transplantation.

	**T cell subset**	**p-p38MAPK**		**p-ERK**		**p-Akt**	
		*r*_*p*_	*p-value*	*r*_*p*_	*p-value*	*r*_*p*_	*p-value*
TAC	CD4+CD28+	−0.09	0.79	0.11	0.73	−0.08	0.82
	CD8+CD28+	0.09	0.78	−0.14	0.66	0.20	0.54
	CD8+CD28−	−0.02	0.96	−0.15	0.64	0.20	0.53
MPA (TAC-group)	CD4+CD28+	−0.40	0.20	0.46	0.13	0.31	0.33
	CD8+CD28+	−0.37	0.24	0.32	0.31	0.37	0.23
	CD8+CD28−	−0.26	0.41	0.31	0.32	0.36	0.25
MPA (BELA-group)	CD4+CD28+	−0.51	0.16	−0.38	0.32	−0.28	0.47
	CD8+CD28+	−0.60	0.09	−0.17	0.66	−0.32	0.39
	CD8+CD28−	−0.29	0.44	−0.08	0.85	−0.35	0.35

r_p_ Pearson correlation coefficient.

### Association between phosphorylation patterns and acute rejection episodes of BELA-treated patients

**Figure 4:**
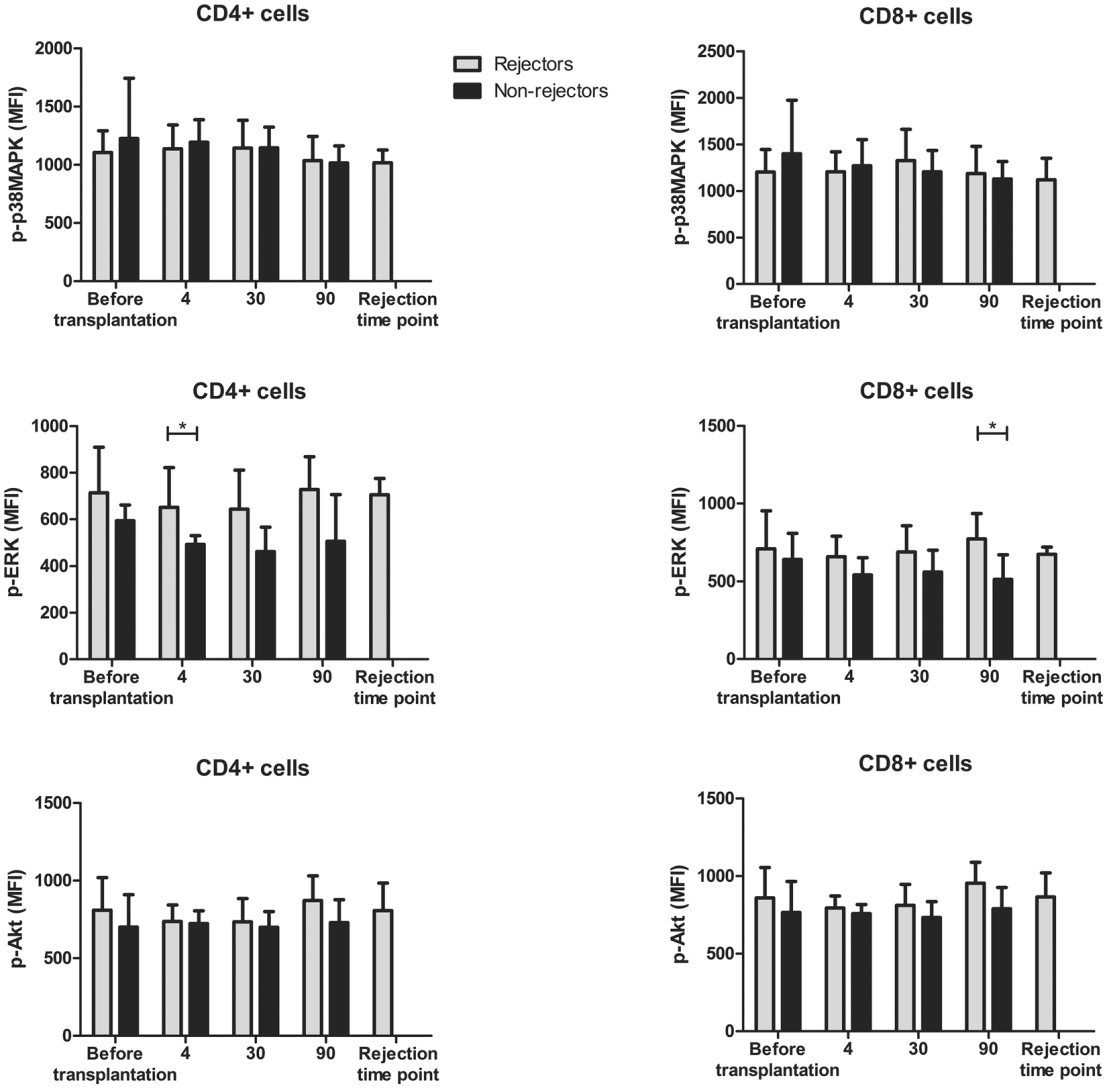
Signaling protein phosphorylation in BELA-treated patients with and without BPAR. Phosphorylation of p38MAPK (upper graphs), ERK (middle graphs) and Akt (lower graphs) in CD4^+^ cells (left) and CD8^+^ cells (right) after stimulation with PMA/ionomycin is shown. BELA-treated patients suffering from an acute rejection (*grey*) episode showed a significantly higher expression of p-ERK before their rejection time point compared to patients without a rejection within 90 days after transplantation (*black*). (Data are plotted as mean ± SD; n = 11 rejectors and n = 9 non-rejectors) *p < 0.05.

The association between TAC-based therapy and the incidence of BPAR could not be analyzed since only two out of 20 TAC-treated patients suffered from an acute rejection. These patients suffered from a Banff type 1 and 2 rejection, respectively^34^. To investigate whether a rejection episode was associated with a change in phosphorylation status, blood samples of BELA-treated patients suffering from BPAR were compared with those who remained rejection-free. Eleven out of 20 BELA-treated patients experienced BPAR^34^. Of these, 1 patient was graded Banff type 1, 8 patients were graded Banff type 2, 1 patient was graded Banff type 3, and 1 patient suffered from a mixed type rejection. Both CD4^+^ and CD8^+^ T cells of these patients revealed an increase in p-ERK expression at day 4 and day 90, respectively, after stimulation with PMA/ionomycin (p < 0.05) (Fig. 4, middle). In addition, p-ERK expression at the time of rejection was high compared to patients without a rejection. There was no difference in p38MAPK and Akt phosphorylation between rejectors and non-rejectors in both cell subsets.

### Cytokine production and phosphorylation of p38MAPK

**Figure 5:**
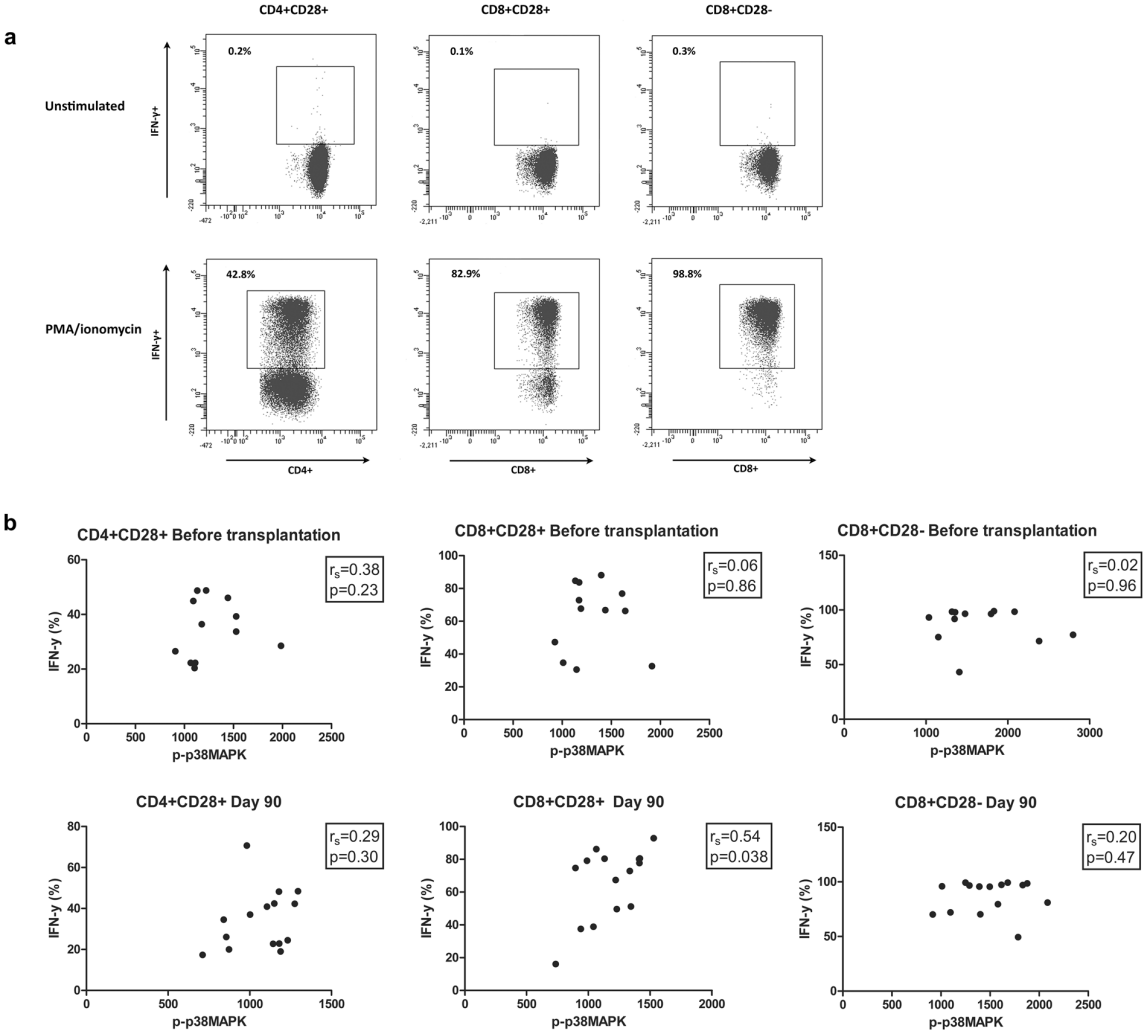
Correlation between signaling protein phosphorylation and IFN-γ production by T cell subsets. (**a**) Gating strategy for the production of IFN-γ by CD4^+^CD28^+^ (left), CD8^+^CD28^+^ (middle) and CD8^+^CD28^−^ (right) cells, in both unstimulated and stimulated samples. (**b**) Correlations between p-p38MAPK (upper graphs), p-ERK (middle graphs) and p-Akt (lower graphs) and the production of IFN-γ by CD4^+^CD28^+^ (left), CD8^+^CD28^+^ (middle) and CD8^+^CD28^−^ (right) cells after stimulation with PMA/ionomycin. Correlations were calculated before transplantation and 90 days after transplantation. P-p38MAPK significantly correlated with the production of IFN-γ in CD8^+^CD28^+^cells at day 90 after transplantation. (n = 12 before transplantation and n = 15 90 days after transplantation); *p < 0.05, **p < 0.01, ***p < 0.001.

To determine whether the pharmacodynamic drug effects also correlated with T cell subset function, correlations were calculated between p-p38MAPK expression and the production of IFN-γ. Before transplantation, no correlation existed between p-p38MAPK expression and IFN-γ production in all tested T cell subsets. At day 90 after transplantation, a significant positive correlation was found between p-p38MAPK expression and IFN-γ production in both CD4^+^CD28^+^ and CD8^+^CD28^+^ T cells (Fig. 5).

## Discussion

Pharmacokinetic monitoring of immunosuppressive drug therapy is routinely performed in most transplant centers. However, the TAC pre-dose concentration does not accurately predict the occurrence of acute rejection after kidney transplantation^2,6^. Here, phospho-specific flow cytometry, a promising technique with a short turnaround time for the pharmacodynamic measurement of immunosuppressive drug effects, was investigated^15,16,41^. The technique has been used previously for testing immunosuppressive drug responses in patients with rheumatoid arthritis and for the monitoring of mTOR inhibitor therapy. This technique was also studied with regard to TDM of TAC by our group^28^. However, and in contrast to the present work, in that study, only p38MAPK was investigated and only in CD4^+^ and CD8^+^ T cells of patients treated with TAC-based immunosuppression. Here, three major signaling molecules involved in T cell activation, p38MAPK, ERK and Akt, were tested as potential biomarkers for detecting biological drug effects of TAC in different T cell subsets and compared to a group of patients treated with BELA-based treatment.

In directly measured blood samples of patients treated with a TAC-based immunosuppressive regimen, expression of p-ERK in CD4^+^ T cells was decreased at almost all measured time points after transplantation, whereas no effect was found on p38MAPK and Akt phosphorylation. In contrast, CD8^+^ T cells showed no decrease in p-ERK but showed a lower expression of p-p38MAPK and p-Akt at day 30.

There was no decrease in p-ERK induced by TAC-based treatment after stimulation with PMA/ionomycin, suggesting that the ERK pathway can still be activated after transplantation, despite the inhibited expression in unstimulated samples. A TAC-based-treatment also affected the expression of p-p38MAPK and p-Akt in stimulated CD4^+^CD28^+^, CD8^+^CD28^+^ and CD8^+^CD28^−^ cells, but not to the same extent. For example, CD8^+^ cells did not show an inhibition of p38MAPK at day 90, while this molecule was significantly inhibited at all measured time points in CD4^+^ cells. P-Akt showed no significant inhibition at day 180 in all T cells subsets.

BELA blocks the co-stimulation signal between the CD80/86 molecule on APC’s and the CD28 molecule on T cells. This indicates that BELA only indirect affects the phosphorylation of signaling pathways in T cells. Indeed, no effects of BELA on signaling protein phosphorylation were noticed in both unstimulated and stimulated samples. Only at day 90, when blood samples were stimulated with PMA/ionomycin, a decrease of p-p38MAPK was found in CD28^+^ cells after treatment with BELA. This effect was not noticed in CD8^+^CD28^−^ cells, indicating that BELA did not affect these cells. However, this effect was not observed at other time points, indicating that any difference between the effect of BELA on CD28^+^ and on the more aggressive CD28^−^ T cells could not be detected with phospho-specific flow cytometry. Notably and in contrast to the TAC-treated study population, expression of p-ERK was increased at day 360 in the presence of BELA, reflecting immune activation.

The absence of decreased signaling protein phosphorylation during BELA-based therapy also suggests that p-p38MAPK and p-Akt are mainly affected by TAC. However, the significant reductions that were found during TAC-based treatment were small and no correlations were found between TAC pre-dose concentrations and p-p38MAPK. However, there could be a correlation between phosphorylation expression and peak drug concentrations or area under the concentration-*versus* time curve, but these were not measured in this study. No other patient baseline characteristic showed an association with the expression of p-p38MAPK, p-ERK or p-Akt, suggesting that the decrease in phosphorylation was not influenced by these parameters. Downstream of the MAPK pathway, p-p38MAPK will initiate the transcription of the IFNG gene, which in turn will lead to the production of IFN-γ by T cells^42,43^. Here, the expression of p-p38MAPK, measured by phospho-specific flow cytometry, significantly correlated with IFN-γ production in CD8^+^CD28^+^ T cells but only after transplantation when there is less variation in p-p38MAPK expression due to the presence of immunosuppressive drugs. Unfortunately, IFN-γ production was not measured at day 4 and 30 after transplantation, but the immunosuppressive drug concentrations were higher at day 90 compared to day 180 and 360 after transplantation suggesting that a TAC-based immunosuppressive drug therapy is involved in the regulation of p38MAPK phosphorylation. Altogether, the correlation between p-p38MAPK and the production of IFN-γ suggests that measuring phosphorylation of p38MAPK could be an effective manner to monitor CD8^+^CD28^+^ T cell function after transplantation.

Eleven out of 20 BELA-treated patients in this study suffered from an acute rejection^34^. BELA-treated patients suffering from a BPAR showed an increased phosphorylation of ERK after stimulation with PMA/ionomycin in both CD4^+^ and CD8^+^ T cells, in contrast to patients without an acute rejection episode. This observation was made at day 4 in CD4^+^ and at day 90 in CD8^+^ cells before the rejection episode was detected. p-ERK could be inhibited in the presence of a TAC-based regimen, as was measured in unstimulated samples. In contrast, a BELA-based treatment even increased the expression of p-ERK at day 360 after stimulation of the samples. Previously, a weak correlation was also found between the expression of p-ERK and antibody-mediated rejection biopsies of heart transplant patients^44^. Moreover, graft survival could be prolonged by the use of an ERK1/2 inhibitor as was shown in a mouse model^45^. Altogether, these results indicate an important role for ERK in both CD4^+^ and CD8^+^ T cells before an acute rejection episode is diagnosed clinically. Unfortunately, it could not be verified in this study whether the expression of p-ERK during a rejection episode was also increased in the presence of a TAC-based treatment, since only two patients in this treatment group suffered from acute rejection.

This study has other limitations, such as the small sample size and the lack of correlations between the expression of p-p38MAPK, p-ERK and p-Akt and clinical outcomes. Larger prospective cohort studies should focus on the phosphorylation of ERK in CD4^+^ and CD8^+^ T cells after transplantation to prove that p-ERK is a reliable biomarker for rejection in the presence of either a BELA- or TAC-based therapy. Such a study should also measure the total expression of ERK to ensure that any effects are due to the phosphorylation inhibition and not to the changed expression of the total protein. Moreover, in order to use phospho-specific flow cytometry for daily clinical diagnostics, the technique should be validated by using area-under the concentration *versus* time-curve values instead of pre-dose concentrations. These values could also give an explanation for the low inhibitory effects on signaling molecule phosphorylation at specific time points and the increase in p-ERK in BELA-treated patients suffering from a rejection.

In conclusion, phospho-specific flow cytometry is a promising technique to monitor the pharmacodynamic effects of a TAC, but not BELA, -based immunosuppressive therapy after transplantation. In contrast to TAC-based treatment, BELA-based immunosuppression does not inhibit key T cell activation pathways, despite the expression of CD28, which may contribute to the high rejection incidence observed among BELA-treated kidney transplant recipients.

## Material and Methods

### Kidney transplant patients

Between January 21^th^_,_ 2014 and February 19^th^, 2016 peripheral blood samples were collected from renal transplant recipients to determine the effect of a TAC- or BELA-based treatment on CD3^+^ T cell subset activation. This study was part of a randomized-controlled clinical trial that was approved by the Medical Ethical Committee of the Erasmus MC, University Medical Center Rotterdam (MEC-2012-421, EudraCT #2012-003169-16, http://www.trialregister.nl/trialreg/index.asp number NTR4242, registered October 17^th^ 2013)^34^. Detailed information about study design, interventions, outcomes, sample size and randomization was previously reported by de Graav *et al*.^34^. All patients (≥18 years) described in this single center study received a kidney from a living donor at our institution (Erasmus MC, Department of Internal Medicine, Rotterdam, the Netherlands)^34^. Kidneys were either allocated directly or as part of the Dutch national kidney exchange program^46^. Recipients of a deceased donor kidney were not included in this study and no organs were procured from (executed) prisoners. All experiments were performed in accordance with relevant guidelines and regulations. Forty renal transplant patients were included and randomized to receive a BELA-based treatment (n = 20) or TAC-based treatment (n = 20). In- and exclusion criteria were described previously^34^. All participants gave written informed consent for participation in this study and for collecting their blood samples. On the day of transplantation and on day 4 after transplantation, patients were treated with 20 mg basiliximab intravenously (Simulect®, Novartis, Basel, Switzerland). During the first three post-operative days prednisolone was administered intravenously in a dosage of 100 mg/day. Afterwards, prednisolone was given orally in a dose of 20 mg and tapered to 5 mg/day by month 3 and was then continued throughout the first post-transplant year. Mycophenolate mofetil (MMF; Cellcept®; Roche, Basel, Switzerland) was given in a starting dose of 2000 mg/day equally divided in two doses, and then adjusted to pre-dose concentrations (target 1.5–3.0 mg/L). Patients received TAC (Prograf®, Astellas Pharma Inc., Tokyo, Japan) twice a day from the day of transplantation with a starting dose of 0.2 mg/kg/day. Thereafter, TAC was adjusted to pre-dose concentrations: 10–15 ng/mL (week 1–2), 8–12 ng/mL (week 3–4), and 5–10 ng/mL (from week 5 onwards). BELA-treated patients received 10 mg/kg/day BELA (Nulojix®, Bristol-Myers Squibb, New York, USA) intravenously on the day of transplantation and on days 4, 15, 30, 60, and 90 after transplantation. From month 4 onwards, patients received monthly infusions of 5 mg/kg BELA (according to the so-called less intensive regimen)^33^. Heparinized blood samples were collected pre-transplantation and 4, 30, 90, 180 and 360 days post-transplantation or before anti-rejection therapy was started when an acute rejection was suspected (usually on the day of the kidney biopsy).

### Immunosuppressive drug whole-blood pre-dose concentrations

TAC and MPA whole-blood or plasma pre-dose concentrations, respectively, were determined in EDTA blood using the antibody-conjugated magnetic immunoassay on a Dimension Xpand analyzer (Siemens HealthCare Diagnostics Inc., Newark, DE) according to the manufacturer’s instructions. The lower and upper limits of detection of TAC were 1.5 and 30 ng/mL and for MPA 0.5 μg/ml and 15 μg/ml, respectively. For TAC, the coefficient of variations (CV) was 15.0%, 8.9% and 11.2% for the low, middle and high control samples, respectively. For MPA, the CV was 3.9% and 3.7%, for the low and high controls, respectively. Proficiency samples were obtained from the UK Quality Assessment Scheme (Analytical Services International Ltd, London, UK) and the laboratory successfully participates in international proficiency testing schemes.

### Whole-blood phospho-specific flow cytometry

Whole-blood samples were monitored for the expression of phosphorylated (p-) p38MAPK, ERK and Akt according to the manufacturer’s instructions for phospho-protein analysis (BD Biosciences, San Jose, CA) and as described previously^47^. The CV for phospho-specific flow cytometry was 5.6%^48^. In short, 200 μl heparinized blood was stained with Brilliant Violet (BV) 510-labeled mouse anti-human CD3 (Biolegend, San Diego, CA), peridinin chlorophyll (PERCP)-labeled mouse anti-human CD4 (BD Biosciences), allophycocyanin(APC)-Cy7-labeled mouse anti-human CD8 (Biolegend), BV421-labeled mouse anti-human CD28 (BD Biosciences) and Fluorescein Isothiocyanate (FITC)-labeled mouse anti-human CD14 (Serotec, Oxford, UK) for 30 minutes at 37 °C. After 15 minutes of staining, PMA/ionomycin (Sigma-Aldrich, Steinheim, Germany) was added to activate T cells in the remaining 15 minutes. A final optimized concentration of 500 ng/ml and 5 μg/ml of PMA and ionomycin, respectively was used for samples stained for p38MAPK and Akt. 100 ng/ml PMA and 1 μg/ml ionomycin was used for ERK. From here, unstimulated and stimulated cells with PMA/ionomycin were considered as two different samples and analyzed separately. Cells were then fixed and lysed for 10 minutes with Lyse/Fix buffer (BD Biosciences) and permeabilized with 90% methanol for 30 minutes at −20 °C. Phycoerythrin (PE)-labeled mouse anti-p-p38MAPK (clone pT180/pY182), PE-labeled mouse anti-p-Akt (clone pS473) or AlexaFluor647 (AF647)-labeled mouse anti-p-ERK1/2 (pT202/pY204) mAB (all from BD Biosciences) were used for 30 minutes at room temperature to stain for the intracellular signaling pathway activation. Samples were analyzed on a FACS Canto II flow cytometer (BD Biosciences). Isotype controls; mouse IgG1-PE (p38MAPK and Akt, Biolegend) and mouse IgG1-AF647 (ERK; Biolegend); served as negative controls and were included in separated tubes. Cytocalbeads (Thermo Scientific, Fremont, CA) were used to correct for interday-variability of the flow cytometer according to the manufacturer’s instructions. Absolute numbers of CD3^+^, CD4^+^ and CD8^+^ cells were measured with BD multi-test 6-colour in BD TruCount Tubes (BD Biosciences, San Jose, CA).

### Cytokine production

Heparinized blood samples were activated with a final concentration of 0.5 μg/ml PMA and 10 μg/ml ionomycin for four hours at 37 °C. Golgiplug (BD Biosciences) was added during this incubation to accumulate cytokines intracellularly. Subsequently, EDTA was added for 15 minutes. Cells were then stained with BV510-labeled mouse anti-human CD3 (Biolegend), BV421-labeled mouse anti-human CD4 (Biolegend), APC-Cy7-labeled mouse anti-human CD8 (Biolegend) and PERCP-Cy5-labeled mouse anti-human CD28 (BD Biosciences) for 30 minutes at room temperature, fixed twice for 10 minutes with FACS lysing solution (BD Biosciences) and treated with permeabilization buffer II (BD Biosciences) for 10 min. FITC-labeled mouse anti-human IFN-γ (BD Biosciences) was used for intracellular cytokine staining for 30 minutes at room temperature.

### Statistical Analysis

The Median Fluorescence Intensity (MFI) was measured for the phosphorylation of p38MAPK, ERK and Akt and data-analysis was performed with Diva-version 6.0 software (BD Bioscience). Negative values measured with flow cytometry can be explained by the compensation settings of the FACS and are displayed via hyperlog transformation^49^. MFI values were normalized using Cytocalbeads (Thermo Scientific). Statistical analysis was performed with Graph Pad Prism 5.0 (Graph Pad Software Inc., La Jolla, CA) by using paired and unpaired t-tests (after finding a p-value > 0.05 with the Kolmogorov-Smirnov test for normality for the study population). Correlations between drug concentrations and phosphorylation were calculated as the Pearson correlation coefficient. Associations between phosphorylation levels and co-variates were tested by linear regression with IBM SPSS statistics software (version 21; IBM Analytics, Chicago, Illinois, USA). Bonferroni correction was used to correct for multiple testing. A two-sided p-value < 0.05 was considered statistically significant and for the association calculations, two-sided p-values of <0.0042 were considered statistically significant after Bonferroni correction.

### Data availability

All data generated or analyzed during this study are included in this published article and its Supplementary Information files.

## Electronic supplementary material

Supplemenatry Figures and Tables
