# Supplementary material for: Differential T Cell Signaling Pathway Activation by Tacrolimus and Belatacept after Kidney Transplantation: Post Hoc Analysis of a Randomised-Controlled Trial
Source: Sci Rep. 2017 Nov 9;7:15135. doi: 10.1038/s41598-017-15542-y (PMC5680251; doi:10.1038/s41598-017-15542-y)
Supplement: Supplementary file 1 — Supplemenatry Figures and Tables [file 41598_2017_15542_MOESM1_ESM.pdf]

Supplementary Figures and Tables

Differential T Cell Signaling Pathway Activation by Tacrolimus and Belatacept after Kidney Transplantation: Post Hoc Analysis of a Randomised-Controlled Trial

NYNKE M. KANNEGIETER*; DENNIS A. HESSELINK; MARJOLEIN DIETERICH; GRETCHEN N. DE GRAAV; RENS KRAAIJEVELD; CARLA C. BAAN

*corresponding author

Department of Internal Medicine, Erasmus MC, University Medical Center Rotterdam, Rotterdam, the Netherlands

Address for Correspondence:

Nynke M. Kannegieter

Dept. of Internal Medicine, Division of Nephrology and Transplantation

Erasmus MC, University Medical Center Rotterdam

Room Na-522

Wytemaweg 80

3015 CN, Rotterdam

The Netherlands

E-mail: n.kannegieter@erasmusmc.nl

Phone: +31-(0)10-7033867

Supplementary Figure S1:

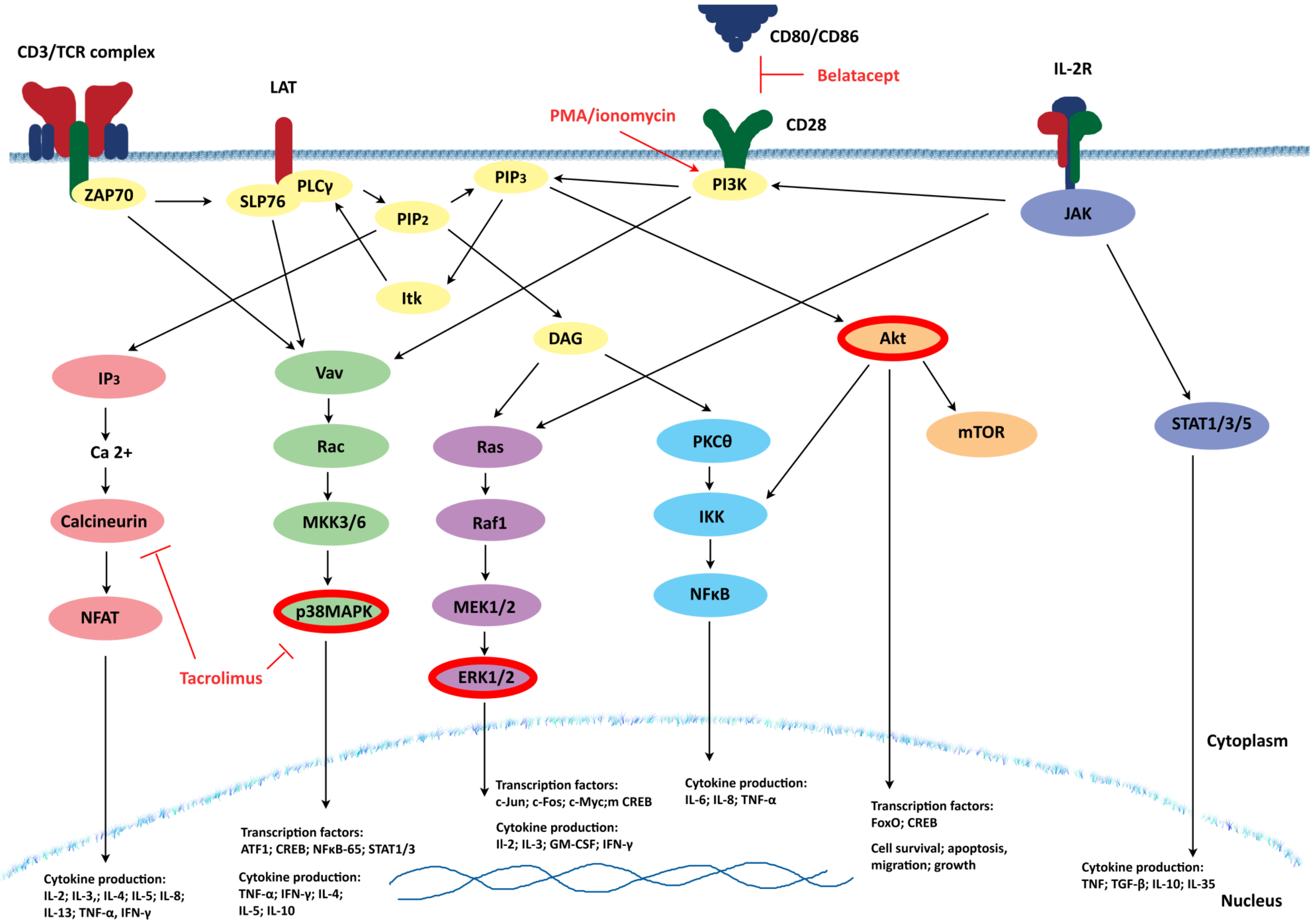

Supplementary Figure S1. Schematic overview of T cell signaling pathways and their interaction downstream of the TCR. After antigen recognition by the TCR and after receiving a co-stimulatory signal via CD28, several intracellular signaling pathways become activated via phosphorylation. These pathways include the signaling molecules p38MAPK, ERK1/2 and Akt. Downstream of the signaling pathways, this activation will initiate gene-transcription and the control of T cell functions, such as cytokine production, cell survival, cell differentiation and cell apoptosis. Activation of the signaling pathways can be mimicked by the stimulation of the T cell with PMA/ionomycin. TAC is known to inhibit the calcineurin and p38MAPK pathway, while BELA blocks the co-stimulatory signal between the CD80/86 molecule on APC's and the CD28 molecule on T cells.

Supplementary Table S2: Baseline characteristics at time of transplantation*

	Belatacept group (n = 20)	Tacrolimus group (n = 20)	p
Age (years)	57 (25-76)	55 (21-76)	0.88
Male / female	14 (70%) / 6 (30%)	16 (80%) / 4 (20%)	0.72
Ethnicity			1.00
• Caucasian	17 (85%)	16 (80%)	
• African	2 (10%)	2 (10%)	
• Asian	1 (5%)	2 (10%)	
Body weight (kg)	79.0 (56.6-111.4)	93.6 (51.4-120.0)	0.06
HLA A mismatch (mean ± SD)	1.1 (± 0.7)	1.4 (± 0.5)	0.13
HLA B mismatch (mean ± SD)	1.3 (± 0.5)	1.5 (± 0.5)	0.51
HLA DR mismatch (mean ± SD)	1.1 (± 0.4)	1.3 (± 0.4)	0.70
Current PRA (%)	0 (0-5)	0 (0-17)	0.30
Peak PRA (%)	4 (0-6)	4 (0-21)	0.78
CMV status			0.80
• Donor + / Recipient -	3 (15%)	2 (10%)	
• Donor + / Recipient +	4 (20%)	7 (35%)	
• Donor - / Recipient -	7 (35%)	6 (30%)	
• Donor - / Recipient +	6 (30%)	5 (25%)	
Donor age (years)	59 (24-71)	51 (22-80)	0.18
Related / unrelated donor	6 (30%) / 14 (70%)	5 (25%) / 15 (75%)	1.00
Cause of end-stage renal disease			0.09
• Diabetes mellitus	3 (15%)	7 (35%)	
• Hypertension	2 (10%)	5 (25%)	
• IgA nephropathy	1 (5%)	3 (15%)	
• Polycystic kidney disease	3 (15%)	3 (15%)	
• Obstructive nephropathy	3 (15%)	1 (5%)	
• Unknown	5 (25%)	0 (0%)	
• Other	3 (15%)	1 (5%)	
Renal replacement therapy			0.91
• None (pre-emptive)	10 (50%)	12 (60%)	
• Hemodialysis	7 (35%)	6 (30%)	
• Peritoneal dialysis	3 (15%)	2 (10%)	
Time on dialysis (days)	425 (123-2782)	605 (465-1519)	0.41
Number of kidney transplantation			1.00
• First	19 (95%)	20 (100%)	1.00
• Second	1 (5%)	-	

Continuous variables are presented as medians (plus ranges) and categorical variables as numbers (plus percentages), unless otherwise specified

BPAR, biopsy-proven acute rejection; CMV, cytomegalovirus; HLA, human leukocyte antigen; PRA, panel reactive antibodies (current = PRA at time of transplantation, peak = historically highest measured PRA); SD, standard deviation.

Supplementary Table S3: Incidence of rejection according to the treatment group*

	**Belatacept group (n = 20)**	**Tacrolimus group (n = 20)**	**p**
Borderline	0 (0%)	0 (0%)	-
Type 1			1.00
• 1A	0 (0%)	0 (0%)	
• 1B	1 (5%)	1 (5%)	
Type 2			0.004
• 2A	2 (10%)	1 (5%)	
• 2B	6 (30%)	0 (0%)	
Type 3	1 (5%)	0 (0%)	1.00
Mixed	1 (5%)	0 (0%)	1.00
Total BPAR	11 (55%)	2 (10%)	0.006

The incidence of the first rejection episodes is given. The highest Banff score is depicted if sequential biopsies were performed.

BPAR, Biopsy-proven acute rejection

Supplementary Table S4: Immunosuppressive drugs dose and pre-dose concentrations

	TAC-treated patients					*BELA-treated patients*			
	**TAC dose** (mg)	**TAC C_0** (ng/ml)	**MMF dose** (mg)	**MPA C_0** (µg/ml)	**PRED dose** (mg)	**BELA dose** (mg)	**MMF dose** (mg)	**MPA C_0** (µg/ml)	**PRED dose** (mg)
**Day 4**	16.7 (± 2.5)	16.8 (± 6.2)	2000 (± 0)	3.35 (± 1.59)	20.0 (± 0)	792 (± 153)	2000 (± 0)	3.85 (± 1.55)	20.0 (± 0)
**Day 30**	8.9 (± 4.6)	9.8 (± 3.3)	1750 (± 546)	2.69 (± 1.57)	12.5 (± 3.3)	767 (± 106)	1682 (± 603)	3.39 (± 1.74)	10.5 (± 2.7)
**Day 90**	4.7 (± 1.4)	6.8 (± 1.7)	1342 (± 473)	2.83 (± 1.62)	5.4 (± 1.3)	774 (± 130)	1364 (± 393)	3.27 (± 1.57)	5.0 (± 0)
**Day 180**	4.3 (± 1.1)	6.3 (± 1.5)	1181 (± 499)	2.17 (± 0.98)	4.7 (± 1.2)	383 (± 63)	1222 (± 441)	2.15 (± 1.34)	5.0 (± 0)
**Day 360**	4.2 (± 1.3)	7.0 (± 2.3)	1103 (± 493)	2.02 (± 0.92)	4.3 (± 1.5)	382 (± 58)	1139 (± 377)	1.99 (± 0.63)	5.0 (± 0)

mean (±SD)

Supplementary Figure S5

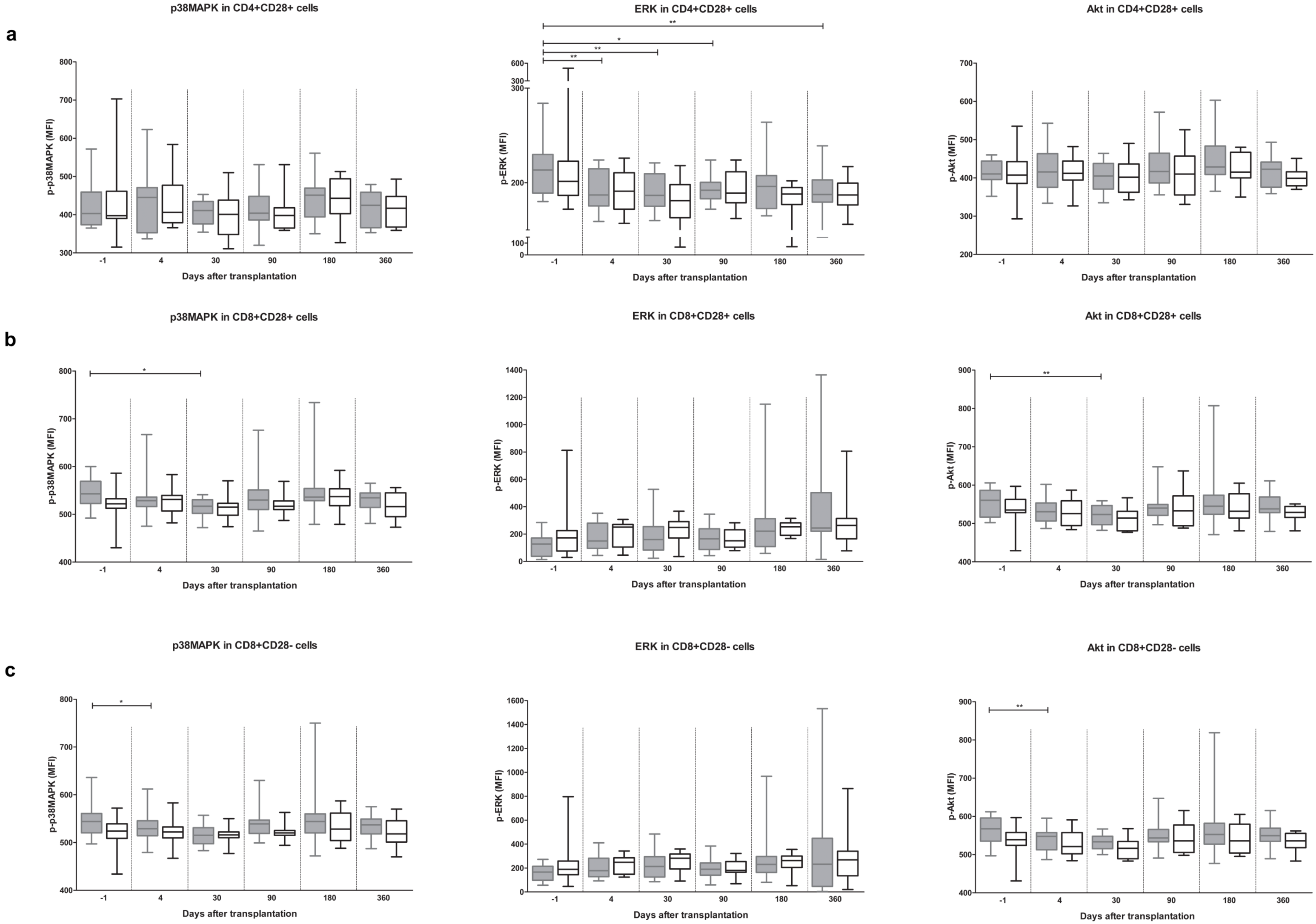

Supplementary Figure S5. P-p38MAPK, p-ERK and p-Akt in unstimulated T cell subsets of TAC (*grey*)- and BELA (*white*)-treated patients before and after transplantation. **a**) p-p38MAPK (left), p-ERK (middle) and p-Akt (right) within $CD4^{+}CD28^{+}$ T cells. P-ERK was significantly decreased in TAC-treated patients, but not in BELA-treated patients. **b**) p-p38MAPK (left), p-ERK (middle) and p-Akt (right) within $CD8^{+}CD28^{+}$ T cells. P-p38MAPK and p-AKT were only inhibited at day 30 after transplantation when TAC was given. **c**) p-p38MAPK (left), p-ERK (middle) and p-Akt (right) within $CD8^{+}CD28^{-}$ T cells. Phosphorylation inhibition is comparable with $CD8^{+}CD28^{+}$ T cells. (Data are plotted as box and whiskers indicating total range; n=20 TAC-treated patients and n=20 BELA-treated patients) *) $p < 0.05$, **) $p < 0.01$, ***) $p < 0.001$

Supplementary Table S6. Univariate regression analysis between patient demographic characteristics and signaling protein phosphorylation

	Predictor	p-p38MAPK		p-ERK		p-Akt	
		β	*p value*	*β*	*p value*	*β*	*p value*
**Before trans-plantation**	Age (years)	0.381	*0.045*	-0.175	*0.374*	0.126	*0.523*
	Gender (male vs female)	-0.267	*0.169*	0.267	*0.169*	-0.273	*0.160*
	Ethnicity (non caucasian vs caucasian)	-0.370	*0.053*	-0.016	*0.934*	-0.324	*0.092*
	Bodyweight before transplantation (kg)	0.222	*0.257*	0.321	*0.096*	0.210	*0.284*
	Highest PRA (%)	0.068	*0.730*	0.049	*0.805*	0.069	*0.727*
	HLA total mismatches (4 or more vs 3 or less)	-0.046	*0.815*	0.102	*0.607*	-0.075	*0.704*
	HLA DR mismatches (2 vs 1)	-0.228	*0.243*	0.197	*0.315*	0.147	*0.457*
	CMV (negative vs positive)	0.234	*0.230*	-0.363	*0.058*	0.142	*0.470*
	Renal replacement therapy (no vs yes)	-0.067	*0.735*	-0.023	*0.906*	-0.099	*0.616*
	CD86 molecules/monocyte pre-transplantation	-0.065	*0.741*	-0.038	*0.847*	-0.101	*0.607*
	Donor age (years)	0.217	*0.267*	-0.100	*0.613*	0.044	*0.823*
	Donor gender (male vs female)	-0.032	*0.873*	-0.072	*0.715*	-0.029	*0.884*
**Day 4**	Age (years)	0.423	*0.031*	0.049	*0.806*	-0.252	*0.205*
	Gender (m vs f)	-0.346	*0.084*	0.021	*0.915*	-0.033	*0.872*
	Ethnicity (non caucasian vs caucasian)	-0.285	*0.159*	-0.006	*0.975*	-0.152	*0.448*
	treatment group (tacrolimus vs belatacept	0.118	*0.566*	-0.134	*0.505*	0.242	*0.225*
	Bodyweight before transplantation (kg)	-0.026	*0.900*	0.253	*0.203*	0.087	*0.667*
	Highest PRA (%)	0.029	*0.890*	-0.203	*0.310*	0.231	*0.246*
	HLA total mismatches (4 or more vs 3 or less)	-0.084	*0.689*	-0.261	*0.188*	-0.247	*0.214*
	HLA DR mismatches (2 vs 1)	-0.349	*0.087*	-0.054	*0.789*	-0.430	*0.025*
	CMV (negative vs positive)	0.270	*0.191*	0.112	*0.577*	0.054	*0.788*
	Renal replacement therapy (no vs yes)	0.087	*0.681*	-0.081	*0.686*	0.057	*0.779*
	CD86 molecules/monocyte pre-transplantation	-0.061	*0.771*	-0.027	*0.895*	-0.144	*0.473*
	Donor age (years)	0.442	*0.027*	-0.239	*0.230*	0.133	*0.510*
	Donor gender (male vs female)	-0.047	*0.823*	-0.092	*0.649*	0.112	*0.577*
**Day 360**	Age (years)	0.388	*0.055*	0.213	*0.307*	0.049	*0.816*
	Gender (m vs f)	-0.213	*0.307*	-0.090	*0.669*	-0.115	*0.586*
	Ethnicity (non caucasian vs caucasian)	-0.007	*0.974*	-0.211	*0.312*	-0.054	*0.797*
	treatment group (tacrolimus vs belatacept	0.180	*0.389*	-0.099	*0.639*	-0.092	*0.662*
	Bodyweight before transplantation (kg)	-0.162	*0.438*	0.161	*0.442*	-0.045	*0.829*
	Highest PRA (%)	-0.131	*0.533*	-0.157	*0.453*	-0.148	*0.481*
	HLA total mismatches (4 or more vs 3 or less)	-0.148	*0.481*	-0.136	*0.518*	-0.281	*0.173*
	HLA DR mismatches (2 vs 1)	0.020	*0.923*	-0.106	*0.613*	0.154	*0.462*
	CMV (negative vs positive)	0.285	*0.167*	0.316	*0.124*	0.346	*0.090*
	Renal replacement therapy (no vs yes)	0.080	*0.705*	0.198	*0.344*	-0.046	*0.828*
	CD86 molecules/monocyte pre-transplantation	0.279	*0.176*	0.171	*0.414*	0.303	*0.141*
	Donor age (years)	0.424	*0.035*	0.369	*0.070*	0.155	*0.458*
	Donor gender (male vs female)	-0.330	*0.107*	0.060	*0.774*	-0.275	*0.184*
